# A Cancer-specific Monoclonal Antibody Recognizes the Aberrantly Glycosylated Podoplanin

**DOI:** 10.1038/srep05924

**Published:** 2014-08-01

**Authors:** Yukinari Kato, Mika Kato Kaneko

**Affiliations:** 1 Department of Regional Innovation, Tohoku University Graduate School of Medicine, 2-1 Seiryo-machi, Aoba-ku, Sendai, Miyagi 980-8575, Japan

## Abstract

Podoplanin (PDPN/Aggrus/T1α), a platelet aggregation-inducing mucin-like sialoglycoprotein, is highly expressed in many cancers and normal tissues. A neutralizing monoclonal antibody (mAb; NZ-1) can block the association between podoplanin and C-type lectin-like receptor-2 (CLEC-2) and inhibit podoplanin-induced cancer metastasis, but NZ-1 reacts with podoplanin-expressing normal cells such as lymphatic endothelial cells. In this study, we established a cancer-specific mAb (CasMab) against human podoplanin. Aberrantly glycosylated podoplanin including keratan sulfate or aberrant sialylation, which was expressed in LN229 glioblastoma cells, was used as an immunogen. The newly established LpMab-2 mAb recognized both an aberrant *O*-glycosylation and a Thr55-Leu64 peptide from human podoplanin. Because LpMab-2 reacted with podoplanin-expressing cancer cells but not with normal cells, as shown by flow cytometry and immunohistochemistry, it is an anti-podoplanin CasMab that is expected to be useful for molecular targeting therapy against podoplanin-expressing cancers.

Podoplanin is a platelet aggregation-inducing type I transmembrane sialoglycoprotein^1^,^2^. Recently, several physiological functions of podoplanin have been reported. Local sphingosine-1-phosphate release after podoplanin-CLEC-2-mediated platelet activation is critical for high endothelial venule integrity during immune responses^3^. The activation of CLEC-2 by podoplanin rearranges the actin cytoskeleton in dendritic cells to promote efficient motility along stromal surfaces^4^. The development of ectopic lymphoid follicles is dependent on IL-17 and Th17-expressing podoplanin^5^. Furthermore, the podoplanin-CLEC-2 interaction is important for platelet aggregation and embryonic blood-lymphatic vascular separation^6^,^7^,^8^. These reports show that podoplanin possesses critical physiological functions. Therefore, inhibiting podoplanin function using anti-podoplanin mAbs might inhibit its critical physiological functions. By contrast, podoplanin expression has been reported in many cancers, including squamous cell carcinomas (head and neck, lung, and esophageal carcinomas), malignant brain tumors, malignant mesotheliomas, bladder cancers, and testicular tumors^1^,^9^,^10^,^11^,^12^,^13^,^14^,^15^,^16^,^17^. Many reports have described that podoplanin expression is associated with malignant progression and cancer metastasis^6^,^15^. Overexpression of podoplanin is reportedly associated with clinical outcomes^12^. Podoplanin has also been reported to be expressed by tumor-initiating cells (TICs)^18^; therefore, immunotherapy using specific antibodies reactive to podoplanin may eliminate TICs in podoplanin-expressing cancers. Because TICs are thought to be responsible for relapse and to be resistant to conventional therapies, targeting TICs may be a promising approach to cancer therapy.

In this study, we developed a cancer-specific mAb (CasMab) against human podoplanin. The newly established LpMab-2 antibody demonstrated dual recognition of aberrant glycosylation and a podoplanin peptide. LpMab-2 reacted with podoplanin-expressing cancer cells but not with normal cells; therefore, LpMab-2 is an anti-podoplanin CasMab that is expected to be useful for molecular targeting therapy against podoplanin.

## Results

### Production of cancer-type podoplanin

**Figure 1:**
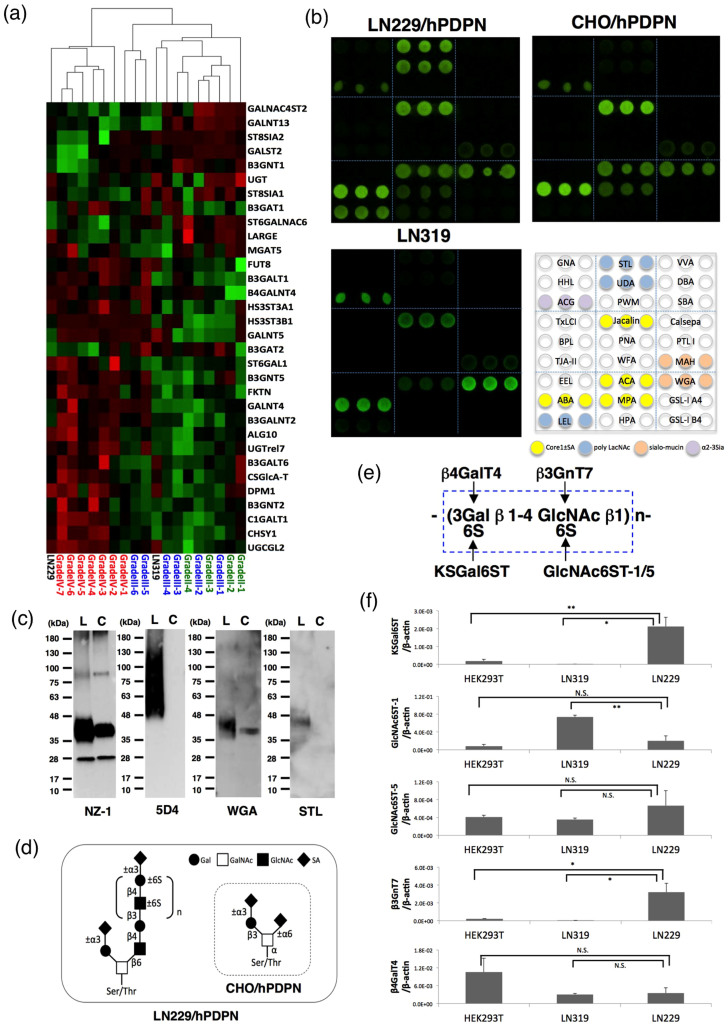
Production of cancer-type podoplanin. (a) Real-time PCR analyses of glycogenes revealed that LN229 cells were clustered into glioblastoma tissues (WHO Grade IV); by contrast, another glioblastoma cell line, LN319, was clustered into WHO Grade II/III. (b) Lectin microarray. Podoplanins on LN229/hPDPN, LN319, and CHO/hPDPN cells were solubilized using PBST. Then, 100 μl of purified podoplanin was applied to the lectin array. After incubation at 20°C for 17 h, the reaction solution was discarded. The glass slide was scanned using a GlycoStation Reader 1200. (c) Western-blot analyses. Purified podoplanin (0.1 μg) was boiled in SDS sample buffer, electrophoresed, and transferred onto a PVDF membrane. After blocking, the membrane was incubated with primary antibodies (NZ-1, 5D4) or biotinylated lectin (WGA, STL) and then with peroxidase-conjugated secondary antibodies or streptavidin-HRP; the membrane was developed with ECL-plus reagents using a Sayaca-Imager. L, LN229/hPDPN; C, CHO/hPDPN. (d) Schematic illustration of the glycan structure of podoplanin. Podoplanin in LN229/hPDPN cells possesses both polylactosamine and sialylated core 1, whereas podoplanin in CHO/hPDPN cells possesses only sialylated core 1. (e) Structure and synthesis of keratan sulfate. (f) Transcript levels for KSGal6ST, GlcNAc6ST-1, GlcNAc6ST-5, β3GnT7, and β4GalT4 genes in each cell line were measured using real-time PCR. Values normalized to the level of β-actin transcripts are presented. The error bars show the standard deviation of three independent experiments. Statistical analysis was performed using Student's *t*-test (*p < 0.05, **p < 0.01).

Real-time PCR analyses of glycogenes revealed that the LN229 cell line was clustered into glioblastoma tissues (WHO Grade IV); by contrast, another glioblastoma cell line, LN319, was clustered into WHO Grade II/III (Figure 1a). Therefore, human podoplanin was transfected into LN229 glioblastoma cells (LN229/hPDPN). Human podoplanins, purified from LN229/hPDPN, LN319, and CHO/hPDPN cells, were applied to a lectin microarray^19^. Figure 1b shows that podoplanins from LN319 and CHO/hPDPN react with sialic acid ± core1 binders (Jacalin, *Agaricus bisporus* agglutinin (ABA), *Amaranthus caudatus* agglutinin (ACA)), sialo-mucin binders (*Maackia amurensis* hemagglutinin (MAH), wheat germ agglutinin (WGA)), and an α2-3 sialic acid binder (*Agrocybe cylindricea* galectin (ACG), which shows a high affinity for α2-3 sialyl lactose and α2-3 sialyl LacNAc, as well as LacNAc, α2-3 core1, and core1^20^). Although podoplanin from CHO/hPDPN reacts with *Maclura pomifera* agglutinin (MPA), podoplanin from LN319 did not. The WGA signal of podoplanin from LN319 is much stronger than that of podoplanin from CHO/hPDPN, although the Jacalin signal of podoplanin from LN319 is much weaker than podoplanin from CHO/hPDPN, indicating that podoplanin from LN319 is highly sialylated compared with podoplanin from CHO/hPDPN. Podoplanin from LN229/hPDPN cells also reacted strongly with sialic acid ± core1 binders or sialo-mucin binders. By contrast, podoplanin from LN229/hPDPN cells reacted with polylactosamine binders (*Lycopersicon esculentum* lectin (LEL), *Solanum tuberosum* lectin (STL), *Urtica dioica* agglutinin (UDA)), although podoplanin from LN319 or CHO/hPDPN cells did not. We next investigated whether the polylactosamine structure detected in podoplanin from LN229/hPDPN cells is highly sulfated KS proteoglycan using an anti-KS mAb, clone 5D4. As presented in Figure 1c, 5D4 detected podoplanin purified from LN229/hPDPN cells, although it did not react with podoplanin purified from CHO/hPDPN cells, indicating that the polylactosamine structure detected in podoplanin from LN229/hPDPN cells is highly sulfated KS. Only *O*-glycan is attached to human podoplanin^21^. Therefore, highly sulfated KS should be attached to the Ser/Thr residues of podoplanin (Figure 1d). We conducted quantitative real-time PCR analysis to compare the respective expression levels of five genes involved in KS synthesis (KSGal6ST, GlcNAc6ST-1/-5, β3GnT7, and β4GalT4) in LN229, LN319, and HEK-293T cells (Figure 1e). LN229 expressed all the genes involved in KS synthesis, especially KSGal6ST and β3GnT7, at higher levels than other cell lines (Figure 1f), indicating that of the cell lines, only the LN229 cells could synthesize KS on podoplanin.

### Establishment of a cancer-specific monoclonal antibody (CasMab) against human podoplanin

**Figure 2:**
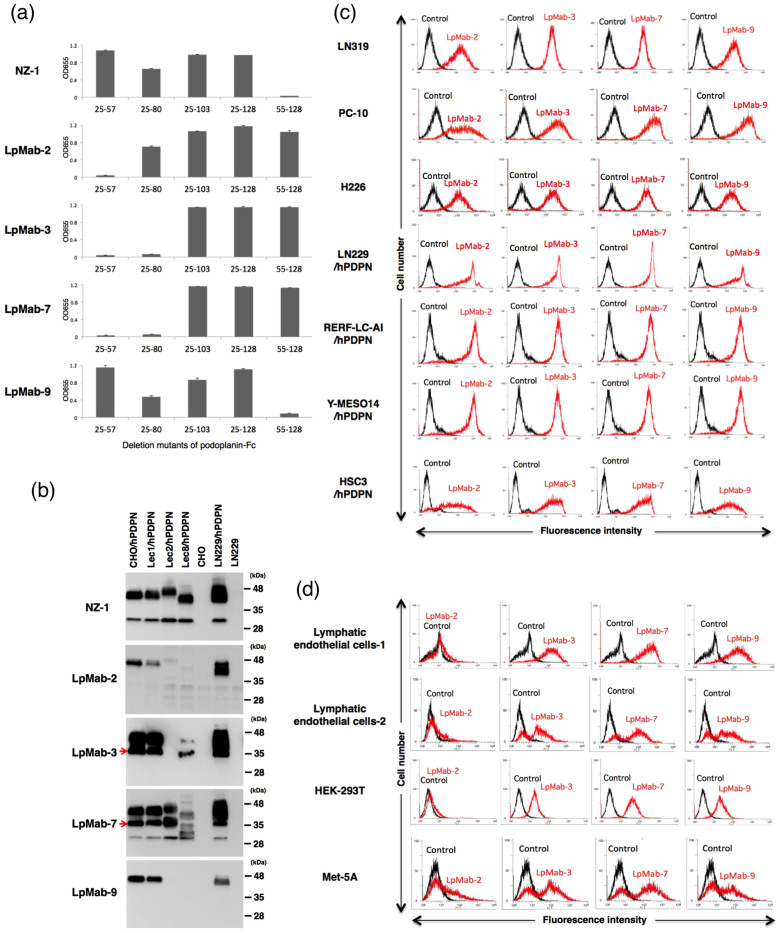
Establishment of a cancer-specific monoclonal antibody (CasMab) against human podoplanin. (a) Epitope mapping of anti-podoplanin mAbs using ELISA. Purified human-podoplanin-Fc chimera proteins were immobilized on Nunc Maxisorp 96-well immunoplates at 1 μg/ml for 30 min. After blocking with SuperBlock T20 (PBS) Blocking Buffer, the plates were incubated with LpMab-2, LpMab-3, LpMab-7, and LpMab-9 (1 μg/ml) followed by 1:1,000 diluted peroxidase-conjugated anti-mouse IgG. The enzymatic reaction was conducted with 1-Step Ultra TMB-ELISA. The optical density was measured at 655 nm using an iMark microplate reader. These reactions were performed in a volume of 50 μl at 37°C. The error bars show the standard deviation of three independent experiments. (b) Characterization of anti-podoplanin mAbs. Cell lysates (10 μg) were boiled in SDS sample buffer, then electrophoresed on 5–20% polyacrylamide gels and transferred onto a PVDF membrane. After blocking, the membrane was incubated with primary antibodies (1 μg/ml and then with peroxidase-conjugated secondary antibodies; the membrane was developed with ECL-plus reagents using a Sayaca-Imager. Arrows indicate a 35-kDa band of podoplanin. (c, d) Flow cytometry using anti-podoplanin mAbs against cancer cells (c) and normal cells (d). Cell lines were harvested by brief exposure to 0.25% Trypsin/1 mM EDTA. After washing with PBS, the cells were treated with primary antibodies (1 μg/ml) for 30 min at 4°C followed by treatment with Oregon green-conjugated anti-mouse IgG. Fluorescence data were collected using a cell analyzer (EC800; Sony Corp.).

To develop novel anti-podoplanin mAbs, we immunized mice with LN229/hPDPN cells, which possess cancer-type glycan patterns, including highly sulfated polylactosamine and aberrant sialylation. The culture supernatants were screened using an enzyme-linked immunosorbent assay (ELISA) for binding to recombinant human podoplanin purified from LN229/hPDPN cells. After limiting dilution of the hybridomas, nine clones were established. Among the nine clones, four clones (LpMab-4, LpMab-5, LpMab-6, and LpMab-8) reacted with not only LN229/hPDPN cells but also LN229 cells, indicating that those four clones detected glycans that are expressed in LN229 cells. Because one clone (LpMab-1) was an IgM class and showed a low binding affinity, the other four mAbs were characterized in further experiments. We performed epitope mapping using several podoplanin-Fc chimera proteins. LpMab-9 (IgG_1_, kappa) reacted with the platelet aggregation-stimulating (PLAG) domain (25–57 amino acids) in the same way as NZ-1 (Figure 2a). The other mAbs, LpMab-2 (IgG_1_, kappa), LpMab-3 (IgG_1_, kappa), and LpMab-7 (IgG_1_, kappa), reacted with a non-PLAG domain. We next performed Western-blot analyses using these mAbs against several glycan-deficient podoplanin transfectants. NZ-1 and LpMab-7 reacted with all the podoplanin transfectants (Figure 2b). By contrast, LpMab-2 and LpMab-9 reacted with CHO/hPDPN, Lec1/hPDPN (*N*-glycan deficient), and LN229/hPDPN cells, but not with Lec2/hPDPN (sialic acid-deficient) or Lec8/hPDPN (*O*-glycan deficient) cells, indicating that the epitopes recognized by LpMab-2 and LpMab-9 include sialylated *O*-glycan-attached podoplanin. LpMab-3 did not react with Lec2/hPDPN cells, although it reacted with the other podoplanin transfectants, indicating that LpMab-3 recognized sialylated podoplanin. Because CHO/hPDPN cells do not express keratan sulfate (Figure 1), the epitope of these mAbs is not keratan-sulfated podoplanin. LpMab-3 and LpMab-7 recognized an additional band of 35 kDa, although the other mAbs did not. In previous studies, no anti-podoplanin mAb has detected this 35-kDa band^22^, indicating that LpMab-3 and LpMab-7 recognize novel unique epitopes.

### Flow cytometric analyses of anti-podoplanin mAbs against podoplanin-expressing cancer cell lines and normal cell lines

We next examined the reactivity of the anti-podoplanin mAbs against several podoplanin-expressing cancer cell lines and normal cell lines using flow cytometry. The novel mAbs reacted with podoplanin-expressing cancer cell lines, including LN319^13^, PC-10^23^, NCI-H226^24^, LN229/hPDPN, RERF-LC-AI/hPDPN, Y-MESO14/hPDPN^16^, and HSC3/hPDPN (Figure 2c). Although LpMab-3, LpMab-7, and LpMab-9 reacted with podoplanin-expressing normal cells, such as lymphatic endothelial cells^7^,^24^, HEK-293T (kidney cells)^25^, and Met-5A (mesothelial cells), the reaction of LpMab-2 with these podoplanin-expressing normal cells was very low (Figure 2d), indicating that LpMab-2 is a CasMab.

### Characterization of the LpMab-2 mAb

**Figure 3:**
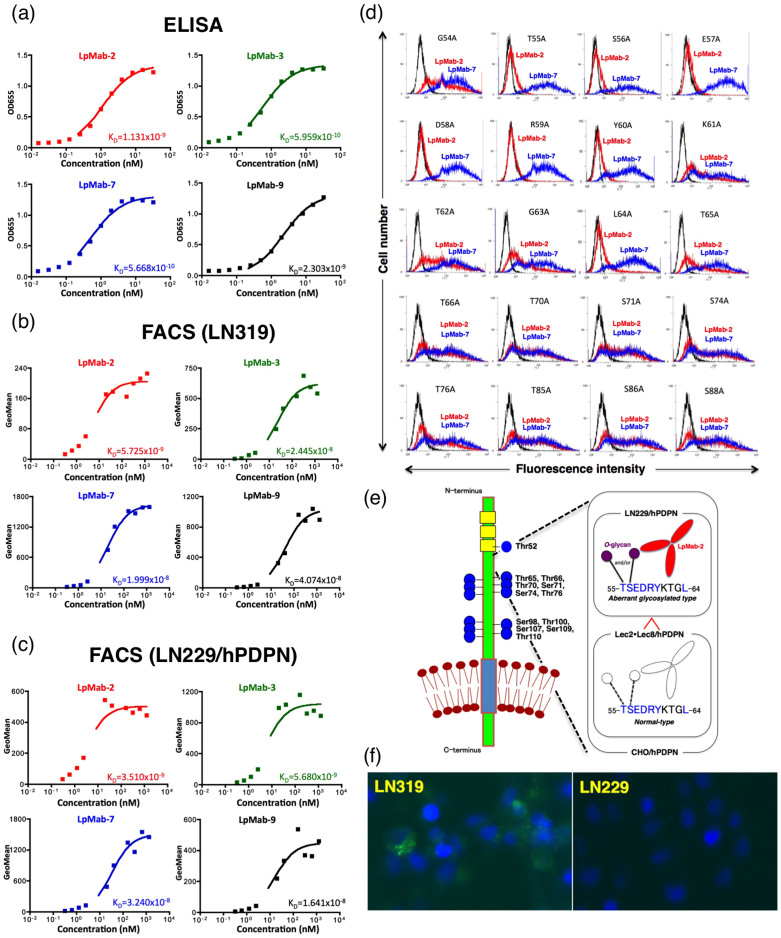
(a) Determination of protein binding affinity using ELISA. The purified human-podoplanin-Fc chimera protein (25 - 128 amino acids of podoplanin + human IgG_1_-Fc) was immobilized at 1 μg/ml. The plates were incubated with serially diluted LpMab-2, LpMab-3, LpMab-7, and LpMab-9 (150 pg/ml–2.5 μg/ml) followed by 1:1,000 diluted peroxidase-conjugated anti-mouse IgG. The dissociation constants (*K_D_*) were obtained by fitting the binding isotherms using the built-in one-site binding models in Prism software. (b, c) Determination of binding affinity using flow cytometry. LN319 or LN229/hPDPN cells (2 × 10^5^ cells) were resuspended with 100 μl of serially diluted LpMab-2, LpMab-3, LpMab-7, and LpMab-9 (0.02–100 μg/ml) followed by secondary anti-mouse IgG. Fluorescence data were collected using an EC800 Cell Analyzer. The dissociation constants (*K_D_*) were obtained by fitting the binding isotherms using the built-in one-site binding models in Prism software. (d) Epitope mapping of LpMab-2 using flow cytometric analyses. Podoplanin mutant-expressing CHO cells were harvested by brief exposure to 0.25% Trypsin/1 mM EDTA. After washing with PBS, cells were treated with LpMab-2 and LpMab-7 (1 μg/ml) for 30 min at 4°C, followed by treatment with Oregon green-conjugated anti-mouse IgG. Fluorescence data were collected using an EC800 Cell Analyzer. (e) Left: The sequencing gap was observed at several Ser/Thr residues in human podoplanin purified from CHO/hPDPN cells by Edman degradation. Well-glycosylated Ser/Thr residues (Thr52, Thr65, Thr66, Thr70, Ser71, Ser74, Thr76, Ser98, Thr100, Ser107, Ser109, and Thr110) were identified. PLAG domains and *O*-glycans are shown in yellow and blue, respectively. Right: Schematic illustration showing dual recognition of LpMab-2 against podoplanin glycopeptide. (f) Internalization assay of LpMab-2 with podoplanin positive-LN319 cells and podoplanin negative-LN229 cells. LN319 and LN229 glioblastoma cells were plated on a 24-well plate and were incubated for 24 h. Then, LpMab-2-pHrodo was added to the medium (30 μg/ml). The cells were incubated for 50 h, then were washed once with PBS. Fluorescence microscopy was performed using a FLoid Cell Imaging Station. The nucleus was stained with DAPI.

Because the binding affinity of antibodies is critical for antibody-based cancer therapy, the dissociation constant (*K_D_*) was next determined using ELISA and flow cytometric analysis^26^. The *K_D_* of LpMab-2 was measured as 1.1 × 10^−9^ M using ELISA and was measured as 5.7 × 10^−9^ M against LN319 cells and 3.5 × 10^−9^ M against LN229/hPDPN cells using flow cytometry (Figure 3a, 3b, 3c). The *K_D_* values of the other mAbs are also shown in Figure 3a, 3b, and 3c. The binding affinity of LpMab-2 was the best of the four mAbs in the flow cytometric analyses (Figure 3b and 3c), although the affinity of LpMab-2 was worse than those of LpMab-3 and LpMab-7 in ELISA (Figure 3a). We next performed a kinetic analysis of the interaction of LpMab-2 with a recombinant podoplanin using surface plasmon resonance (BIAcore)^27^. Determination of the association and dissociation rates from the sensorgrams revealed that a *k*_assoc_ of 3.94 × 10^5^ (mol/L-s)^−1^ and a *k*_diss_ of 4.82 × 10^−3^ s^−1^. The *K*_A_ at binding equilibrium, calculated as *K*_A_ = *k*_assoc_/*k*_diss_, was 8.17 × 10^7^ (mol/L)^−1^, *K*_D_ = 1/*K*_A_ = 1.22 × 10^−8^ M.

The LpMab-2 epitope was determined to be 55–80 amino acids using ELISA (Figure 2a), and sialylated *O*-glycan was included in the LpMab-2 epitope (Figure 2b). Therefore, we produced point mutations at the Ser/Thr residues in amino acids 54–88 of podoplanin (Figure 3d). Of the 12 Ser/Thr residues in this region, the reaction of LpMab-2 against the T55A and S56A mutants was very low, while the other mutants were recognized by LpMab-2, indicating that Thr55 and Ser56 are included in the LpMab-2 epitope. We also produced several point mutations around Thr55 and Ser56. LpMab-2 did not react with the E57A, D58A, R59A, Y60A, or L64A mutants. Taken together, the evidence indicates that the important podoplanin epitope for LpMab-2 is the glycopeptide Thr55-Leu64: *TS*EDRYKTGL (Figure 3e; right). The sequencing gap was observed at several Ser/Thr residues during Edman degradation of podoplanin purified from CHO/hPDPN cells, and well-glycosylated Ser/Thr residues were identified (Figure 3e; left)^21^. The glycosylation of Thr55 and Ser56 was not detected in CHO/hPDPN cells, indicating that only a small population of human podoplanin is glycosylated at Thr55 and Ser56 (Figure 3e; right).

We previously demonstrated that the NZ-1 mAb can be internalized into LN319 glioblastoma cells^27^. Similarly, LpMab-2 was internalized into podoplanin-positive LN319 cells but not into podoplanin-negative LN229 cells (Figure 3f), indicating that LpMab-2 is a candidate for use in an antibody-drug conjugate.

### Immunohistochemical analyses of LpMab-2 against podoplanin-expressing cancers and normal tissues

**Figure 4:**
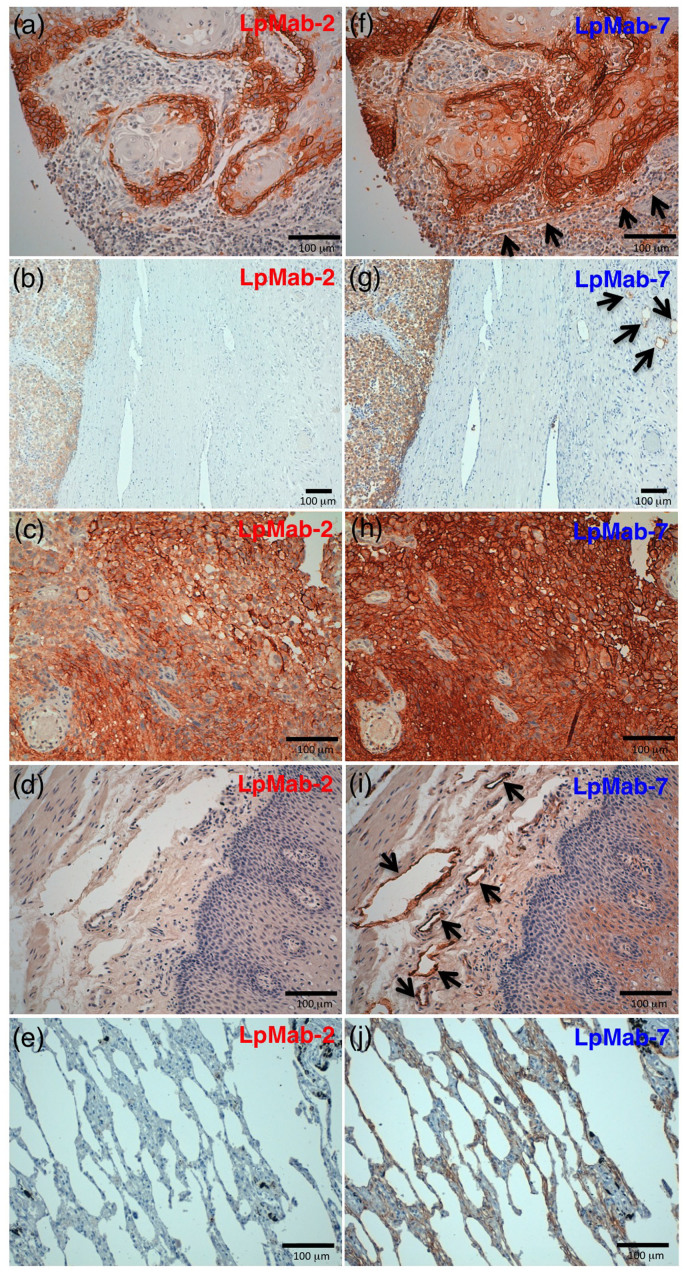
Podoplanin protein expression was determined immunohistochemically in paraffin-embedded tumor specimens. Histologic sections 4 µm thick were deparaffinized in xylene and rehydrated, then autoclaved in citrate buffer (pH 6.0) for 20 min. Sections were incubated with 5 μg/ml of LpMab-2 (a-e) or LpMab-7 (f–j) overnight at 4°C with subsequent treatment using an LSAB kit. Color was developed using 3, 3-diaminobenzidine tetrahydrochloride (DAB) for 10 min and was counterstained with hematoxylin. (a, f) Esophageal squamous cell carcinomas include both cancer cells (upper) and lymphatic endothelial cells (lower). Cancer cells were stained with both LpMab-7 (f) and LpMab-2 (a), whereas lymphatic endothelial cells were stained with LpMab-7 (f), not with LpMab-2 (a). (b, g) Seminoma includes both cancer cells (left) and lymphatic endothelial cells (right, upper). Cancer cells were stained with both LpMab-7 (g) and LpMab-2 (b), whereas lymphatic endothelial cells were stained with LpMab-7 (g), not with LpMab-2 (b). (c, h) Glioblastomas were stained with both LpMab-7 (h) and LpMab-2 (c). (d, i) Esophageal lymphatic endothelial cells were stained with LpMab-7 (i), not with LpMab-2 (d). Lung type I alveolar cells were stained with LpMab-7 (j), not with LpMab-2 (e). Arrows indicate lymphatic endothelial cells (f, g, i).

Because LpMab-2 was determined to be a CasMab by flow cytometry, we performed immunohistochemical analyses of established anti-podoplanin mAbs. LpMab-2 reacted with cancer cells, not with lymphatic endothelial cells in esophageal squamous cell carcinomas (Figure 4a) and seminoma tissues (Figure 4b). By contrast, LpMab-7 stained both cancer cells and lymphatic endothelial cells in esophageal squamous cell carcinomas (Figure 4f) and seminoma tissues (Figure 4g). Both LpMab-2 and LpMab-7 stained glioblastoma cells (Figure 4c and 4h). Immunostaining of LpMab-2 and LpMab-7 against podoplanin demonstrated predominantly cell-surface patterns in cancer cells (Figure 4a, 4c, 4f, 4h). Proliferating endothelial cells were negative for podoplanin (Figure 4c and 4h). LpMab-7 reacted with normal esophageal lymphatic endothelial cells (Figure 4i) and lung type I alveolar cells (Figure 4j), whereas LpMab-2 did not (Figure 4d and 4e), indicating that LpMab-2 also acts as a CasMab in immunohistochemistry. LpMab-3 is also useful for immunohistochemical analyses (data not shown). Not all cancer cells are necessarily aberrantly glycosylated; therefore, the intensity of LpMab-2-staining (Figure 4a, 4b, and 4c) was weaker than LpMab-7 (Figure 4f, 4g, and 4h).

## Discussion

As we previously reported, an anti-podoplanin mAb, NZ-1, was highly internalized into glioma cell lines and also accumulated efficiently into tumors *in vivo*^27^. NZ-1 and its rat-human chimeric anti-podoplanin antibody (NZ-8) possess antibody-dependent cellular cytotoxicity (ADCC) and complement-dependent cytotoxicity (CDC) functions against podoplanin-expressing glioblastoma or malignant mesothelioma cell lines^16^,^26^. Furthermore, NZ-1 inhibited tumor cell-induced platelet aggregation and tumor metastasis by its neutralizing activity^13^, indicating that NZ-1 is a suitable candidate for molecular targeted therapy against podoplanin-expressing cancers. Although many anti-podoplanin mAbs have been produced, those mAbs, including NZ-1, react with podoplanin-expressing normal cells, including lung type I alveolar cells, renal podocytes, mesothelial cells, and lymphatic endothelial cells throughout the body^13^,^22^,^23^,^28^,^29^,^30^. Recently, critical physiological functions of podoplanin have been reported^3^,^4^,^5^,^8^. Therefore, a cancer-specific anti-podoplanin mAb is necessary for molecular targeted therapy against podoplanin-expressing cancers. In our previous study, we revealed that LN229 cells possess cancer-type glycosylation patterns, including highly sulfated keratan sulfate (KS), which are similar to glioblastoma tissues^31^,^32^. Highly sulfated KS, which possesses a polylactosamine structure, was detected in glioblastoma tissues but not in normal brain or low-grade glioma tissues^32^. Therefore, we used a cancer-type podoplanin, which is expressed in LN229/hPDPN cells. Highly sulfated KS may lead to conformational changes in podoplanin because of negative charges; therefore, immunization with highly sulfated KS-possessing podoplanin is a critical method to induce cancer-specific mAbs against podoplanin. Furthermore, aberrant glycosylation, which is observed in LN229/hPDPN cells, may include not only highly sulfated KS but also other several types of glycans, such as aberrant *O*-glycosylation and aberrant sialylation. Although highly sulfated KS was detected by several lectins, such as LEL, STL, and UDA, aberrant *O*-glycosylation or aberrant sialylation were not detected by any lectins using lectin microarray or lectin-blot analyses if they were only attached at different sites with normal type cells and were not over-glycosylated. As we reported previously, Thr55 and Ser56 were non-glycosylated or minimally glycosylated in human podoplanin purified from CHO/hPDPN cells^21^. In addition, LpMab-2 recognized CHO/hPDPN cells at a lower intensity than LN229/hPDPN cells (Figure 2b), indicating that LpMab-2 reacted with cancer-type aberrant glycosylation (*O*-glycosylation or sialylation, not keratan sulfate) of Thr55 and/or Ser56, which is well-glycosylated in LN229/hPDPN cells and partially glycosylated in CHO/hPDPN cells (Figure 3e). Because LpMab-2 reacted with cancer cells and not with lymphatic endothelial cells and type I alveolar cells, LpMab-2 is a cancer-specific mAb. Taken together, the results show that LpMab-2 is expected to be useful for molecular targeting therapy against podoplanin-expressing cancers. We recently produced a single chain Fv fragment (scFv) of an anti-podoplanin mAb (NZ-1) conjugated with immunotoxin, which had an anti-tumor effect in a glioblastoma xenograft model^33^. That report revealed that anti-podoplanin mAbs are effective against glioblastoma after crossing the blood-brain barrier. Likewise, we will perform further experiments using the scFv-immunotoxin of LpMab-2 to investigate its anti-tumor effects against glioblastoma.

## Methods

### Cell lines, animals, and tissue microarrays

All methods were carried out in accordance with the approved guidelines. Chinese hamster ovary (CHO) cells; glycan-deficient CHO cell lines (Lec1, Lec2, and Lec8); and P3U1, Y-MESO-14, HSC3, LN229, HEK-293T, Met-5A, and NCI-H226 cells were obtained from the American Type Culture Collection (ATCC, Manassas, VA). RERF-LC-AI cells were obtained from the RIKEN BioResource Center (Ibaraki, Japan). PC-10 cells were purchased from Immuno-Biological Laboratories Co., Ltd. (Gunma, Japan). Lymphatic endothelial cells-1 and Lymphatic endothelial cells-2 were obtained from Cambrex (Walkersville, MD) and AngioBio (Del Mar, CA), respectively. The human glioblastoma cell line LN319 was donated by Dr. Webster K. Cavenee (Ludwig Institute for Cancer Research, San Diego, CA)^27^. CHO, Lec1, Lec2, Lec8, LN229, Y-MESO-14, RERF-LC-AI, and HSC3 cells were transfected with human podoplanin plasmids (CHO/hPDPN, Lec1/hPDPN, Lec2/hPDPN, Lec8/hPDPN, LN229/hPDPN, Y-MESO-14/hPDPN, RERF-LC-AI/hPDPN, HSC3/hPDPN) using Lipofectamine 2000 (Life Technologies Corp., Carlsbad, CA) or ScreenFect A (Wako Pure Chemical Industries Ltd., Osaka, Japan), according to the manufacturer's instructions^1^,^24^. CHO, Lec1, Lec2, Lec8, P3U1, Y-MESO-14, RERF-LC-AI, HSC3, PC-10, CHO/hPDPN, Y-MESO-14/hPDPN, RERF-LC-AI/hPDPN, and HSC3/hPDPN cells were cultured in RPMI 1640 medium (Wako Pure Chemical Industries, Ltd.) supplemented with 10% heat-inactivated fetal bovine serum (FBS; Life Technologies Corp.), 2 mM L-glutamine (Life Technologies Corp.), 100 units/ml of penicillin, and 100 μg/ml of streptomycin (Life Technologies Corp.) at 37°C in a humidified atmosphere of 5% CO_2_ and 95% air. L-proline (0.04 mg/ml) was added for Lec1, Lec2, and Lec8 cells. LN319, LN229, HEK-293T, and Met-5A cells were cultured in Dulbecco's Modified Eagle's Medium (DMEM) (Wako Pure Chemical Industries Ltd.) supplemented with 10% heat-inactivated FBS, 2 mM L-glutamine, 100 units/ml of penicillin, and 100 μg/ml of streptomycin. MITO + serum extender (Thermo Fisher Scientific Inc., Waltham, MA) was added for Met-5A cells. LEC cells were cultured in endothelial cell medium EGM-2MV supplemented with 5% FBS (Cambrex Corp.). One mg/ml of geneticin (G418; Wako Pure Chemical Industries Ltd.) was added for CHO/hPDPN, Lec1/hPDPN, Lec2/hPDPN, Lec8/hPDPN, LN229/hPDPN, Y-MESO-14/hPDPN, RERF-LC-AI/hPDPN, and HSC3/hPDPN cells. Female BALB/c mice (four-weeks old) were purchased from CLEA Japan (Tokyo, Japan). Animals were housed under pathogen-free conditions. The Animal Care and Use Committee of Tohoku University approved the animal experiments described herein. Tissue microarrays were purchased from Cybrdi, Inc. (Frederick, MD) or BioChain Institute Inc. (Newark, CA).

### Lectin microarray

Podoplanins from LN229/hPDPN and CHO/hPDPN cells were solubilized using 1% Triton-X100 in PBS (PBST) and were purified using a FLAG-tag system (Sigma-Aldrich Corp., St. Louis, MO). Podoplanin from LN319 cells was purified using NZ-1 mAb^21^. Then, 100 μl of purified podoplanin (31.25–2,000 ng/ml) was applied to a lectin array (LecChip ver1.0; GlycoTechnica, Hokkaido, Japan), including triplicate spots of 45 lectins in each of seven divided incubation baths on the glass slide^19^. After incubation at 20°C for 17 h, the reaction solution was discarded. The glass slide was scanned using a GlycoStation Reader 1200 (GlycoTechnica)^13^. Abbreviation of lectins are the following: GNA, *Galanthus nivalis* agglutinin; HHL, *Hippeastrum hybrid* lectin; ACG, *Agrocybe cylindricea* galectin; TxLCI, *Tulipa gesneriana* lectin; BPL, *Bauhinia purpurea alba* lectin; TJA-II, *Trichosanthes japonica* agglutinin; EEL, *Euonymus europaeus* lectin; ABA, *Agaricus bisporus* agglutinin; LEL, *Lycopersicon esculentum* lectin; STL, *Solanum tuberosum* lectin; UDA, *Urtica dioica* agglutinin; PWM, *Pokeweed* mitogen; PNA, *Peanut* agglutinin; WFA, *Wisteria floribunda* agglutinin; ACA, *Amaranthus caudatus* agglutinin; MPA, *Maclura pomifera* agglutinin; HPA, *Helix pomatia* agglutinin; VVA, *Vicia villosa* agglutinin; DBA, *Dolichos biflorus* agglutinin; SBA, Soybean agglutinin; PTL I, *Psophocarpus tetragonolobus lectin* I; MAH, *Maackia amurensis* hemagglutinin; WGA, Wheat germ agglutinin; GSL-I, *Griffonia simplicifolia* lectin I.

### Western-blot analyses

Cell lysates (10 μg) or purified podoplanin (0.1 μg) were boiled in SDS sample buffer (Nacalai Tesque, Inc., Kyoto, Japan)^26^. The proteins were electrophoresed on 5–20% polyacrylamide gels (Wako Pure Chemical Industries Ltd.) and were transferred onto a PVDF membrane (EMD Millipore Corp., Billerica, MA). After blocking with SuperBlock T20 (PBS) Blocking Buffer (Thermo Fisher Scientific Inc.), the membrane was incubated with primary antibodies or biotinylated lectin (1 μg/ml; Vector Laboratories Inc., Peterborough, UK), then with peroxidase-conjugated secondary antibodies (Dako; 1/1,000 diluted) or streptavidin-HRP (Dako; 1/1,000 diluted), and developed with the ECL-plus reagent (Thermo Fisher Scientific Inc.) using a Sayaca-Imager (DRC Co. Ltd., Tokyo, Japan).

### Quantitative real-time PCR analysis

Total RNAs were prepared from glioblastoma cell lines using an RNeasy Plus Mini Kit (Qiagen Inc., Hilden, Germany)^31^. The initial cDNA strand was synthesized using the SuperScript III First-Strand Synthesis System (Life Technologies Corp.) by priming nine random oligomers and an oligo-dT primer, according to the manufacturer's instructions. The cDNAs from glioma tissues (4 diffuse astrocytomas (Grade II), 6 anaplastic astrocytomas (Grade III), 7 glioblastomas (Grade IV)) were synthesized in our previous study^15^. Real-time PCR was performed using CFX Connect (Bio-Rad Laboratories Inc., Philadelphia, PA) with a QuantiTect SYBR Green PCR Kit (Qiagen Inc.). Sets of primers were designed online with Primer3 software. The following oligonucleotides were used: KSGal6ST (forward: TGTTTGAGCCCCTCTACCAC, reverse: GCGGCTTGATGTAGTTCTCC), GlcNAc6ST-1 (forward: AGTTTGCCCTGAACATGACC, reverse: CATGGGCTGGTAGCAAAACT), GlcNAc6ST-5 (forward: CCCCGACGTCTTCTACCTAA, reverse: GCATCAAACACGTCCATGTC), β3GnT7 (forward: CCTCAAGTGGCTGGACATCT, reverse: ACGAACAGGTTTTCCTGTGG), β4GalT4 (forward: AACATCTGCATCCCTTCCTG, reverse: TCATTCTCGGGTACCAGGTC), and β-actin (forward: AGAAAATCTGGCACCACACC, reverse: GGGGTGTTGAAGGTCTCAAA). The PCR conditions were 95°C for 15 min (1 cycle) followed by 45 cycles of 95°C for 5 s, 60°C for 30 s, 72°C for 30 s. Subsequently, a melting curve program was applied with continuous fluorescence measurements. Standard curves for each glycogene and the β-actin template were generated by serial dilution of the PCR products (1 × 10^8^ copies/μl to 1 × 10^2^ copies/μl). The expression level of glycogenes was normalized to the copy number of β-actin. Clustering analysis against glycogenes was performed using the Real Time PCR Clustering Tool (version1.06.00; Research Institute of Bio-System Informatics, Tohoku Chemical Co,. Ltd. Iwate, Japan).

### Hybridoma production

BALB/c mice were immunized by i.p. injection of 1 × 10^8^ LN229/hPDPN cells together with Imject Alum (Thermo Fisher Scientific Inc.). One week later, a secondary i.p. immunization of 1 × 10^8^ LN229/hPDPN cells was performed. After several additional immunizations with 1 × 10^8^ LN229/hPDPN cells, a booster injection was given i.p. 2 days before spleen cells were harvested. The spleen cells were fused with mouse myeloma P3U1 cells using Sendai virus (Hemagglutinating Virus of Japan: HVJ) envelope: GenomONE-CF (Ishihara Sangyo Kaisha, Ltd., Osaka, Japan), according to the manufacturer's instructions. The hybridomas were grown in RPMI medium with hypoxanthine, aminopterin, and thymidine selection medium supplement (Life Technologies Corp.). The culture supernatants were screened using ELISA for binding to recombinant human podoplanin purified from LN229/hPDPN cells (1st screening). Next, flow cytometry was performed against LN229/hPDPN and LN229 cells (2nd screening).

### Expression and purification of soluble podoplanin

cDNAs of human podoplanin containing the extracellular domains of these proteins were obtained by PCR^6^. PCR was performed using HotStarTaq polymerase (Qiagen Inc.). The following oligonucleotides were used: 25–128 (forward: gcgatatcAGAAGGAGCCAGCACAGG, reverse: ggcagatctTGTTGACAAACCATCTTTC), 25–103 (forward (EcoRI-hPod.F1): acgaattcgATGTGGAAGGTGTCAGCTCT, reverse: acagatctGTTTGAGGCTGTGGCGCTTG), 25–80 (forward: EcoRI-hPod.F1, reverse: acagatctGATGCGAATGCCTGTTACAC), 25–57 (forward: EcoRI-hPod.F1, reverse: acagatctTTCGCTGGTTCCTGGAGTCA), 55–128 (forward: EcoRI-hPod.F156, acgaattcAACCAGCGAAGACCGCTATAAGT, reverse: hPod.R384-BglII, acagatctTGTTGACAAACCATCTTTCT). The PCR products were purified, digested with EcoRV or EcoRI and BglII, purified again, and then ligated into the pFUSE-hFc2 (IL2ss) vector (InvivoGen, San Diego, CA), which contains human IgG Fc after the ligation site and interleukin 2 signal sequence (IL2ss) before the ligation site to allow secretion of the Fc-fusion proteins. CHO cells were transfected with the plasmids using the Lipofectamine 2000 (Life Technologies Corp.). For the purification of the fusion proteins, the medium was centrifuged and the obtained supernatant was applied to a Protein G Sepharose 4 Fast Flow column (GE Healthcare, Buckinghamshire, UK). After extensive washing with PBS, the fusion proteins were eluted using 0.1 M glycin and 0.15 M NaCl (pH 2.8), then they were neutralized with 1 M Tris pH 8.0. The proteins were dialyzed against PBS. Expression and purity of the proteins were confirmed by SDS-PAGE.

### Production of podoplanin mutants

The amplified human PDPN cDNA was subcloned into a pcDNA3 vector (Life Technologies Corp.) and a FLAG epitope tag was added at the C-terminus. Substitution of amino acids to alanine in podoplanin was performed using a QuikChange Lightning site-directed mutagenesis kit (Agilent Technologies Inc., Santa Clara, CA)^34^. CHO cells were transfected with the plasmids using a Gene Pulser Xcell electroporation system (Bio-Rad Laboratories Inc.).

### Enzyme-linked immunosorbent assay (ELISA)

Purified proteins were immobilized on Nunc Maxisorp 96-well immunoplates (Thermo Fisher Scientific Inc.) at 1 μg/ml for 30 min^6^. After blocking with SuperBlock T20 (PBS) Blocking Buffer, the plates were incubated with culture supernatant or purified mAbs (1 μg/ml) followed by 1:1,000 diluted peroxidase-conjugated anti-mouse IgG (Dako, Glostrup, Denmark). The enzymatic reaction was conducted with a 1-Step Ultra TMB-ELISA (Thermo Fisher Scientific Inc.). The optical density was measured at 655 nm using an iMark microplate reader (Bio-Rad Laboratories Inc.). These reactions were performed with a volume of 50 μl at 37°C.

### Flow cytometry

Cell lines were harvested by brief exposure to 0.25% Trypsin/1 mM EDTA (Wako Pure Chemical Industries Ltd.)^6^. After washing with phosphate buffered saline (PBS), the cells were treated with primary antibodies (1 μg/ml) for 30 min at 4°C, followed by treatment with Oregon green-conjugated anti-mouse IgG (Life Technologies Corp.). Fluorescence data were collected using a Cell Analyzer EC800 (Sony Corp., Tokyo, Japan).

### Determination of binding-affinity by ELISA

Purified human-podoplanin-Fc chimera protein (25–128 amino acids of podoplanin + human IgG_1_-Fc) was immobilized at 1 µg/ml^26^. The plates were incubated with serially diluted antibodies (150 pg/ml–2.5 μg/ml) followed by 1:1,000 diluted peroxidase-conjugated anti-mouse IgG (Dako). The dissociation constants (*K_D_*) were obtained by fitting the binding isotherms using the built-in one-site binding models in Prism software.

### Determination of the binding affinity by flow cytometry

LN319 cells (2 × 10^5^ cells) were resuspended with 100 μl of serially diluted antibody (0.02–100 μg/ml) followed by secondary anti-mouse IgG (Life Technologies Corp.)^26^. Fluorescence data were collected using a cell analyzer (EC800; Sony Corp.). The dissociation constants (*K_D_*) were obtained by fitting the binding isotherms using the built-in one-site binding models in Prism software.

### Affinity determination by surface plasmon resonance

To determine the affinity, recombinant podoplanin-Fc was immobilized on the surface of chips for analysis using the BIAcore 3000 system (GE Healthcare, Piscataway, NJ). The running buffer was 10 mM HEPES, 150 mM NaCl, and 0.005% v/v Surfactant P20 (pH 7.4; GE Healthcare, BR-1003-68). The mAbs were passed over the biosensor chip, and the affinity rate constants (association rate constant, *k*_assoc_, and disassociation rate constant, *k*_diss_) were determined by nonlinear curve-fitting using the Langmuir one-site binding model of the BIAevaluation software (GE Healthcare). The affinity constant (*K*_A_) at equilibrium was calculated as *K*_A_ = *k*_assoc_/*k*_diss_, and the dissociate constant (*K*_D_) was determined as 1/*K*_A_.

### Internalization assay

LN319 and LN229 glioblastoma cells were plated on a 24-well plate and were incubated for 24 h. LpMab-2 was conjugated with pHrodo Green STP ester (Life Technologies Corp.), according to the manufacturer's instructions^35^. Then, LpMab-2-pHrodo was added to the medium (30 μg/ml). The cells were incubated for 24 h and 50 h. Cells were washed once with PBS, followed by fixation with a 4% paraformaldehyde phosphate buffer solution. Fluorescence microscopy was performed using a FLoid Cell Imaging Station (Life Technologies Corp.). The cell nuclei were stained with DAPI (Life Technologies Corp.).

### Immunohistochemical analyses

Podoplanin protein expression was ascertained immunohistochemically in paraffin-embedded tumor specimens. Briefly, 4-μm-thick histologic sections were deparaffinized in xylene and rehydrated. Then, they were autoclaved in citrate buffer (pH 6.0; Dako) for 20 min. Sections were incubated with 5 μg/ml of primary antibodies overnight at 4°C followed by treatment with an LSAB kit (Dako). Color was developed using 3, 3-diaminobenzidine tetrahydrochloride (DAB; Dako) for 10 min, and then the sections were counterstained with hematoxylin (Wako Pure Chemical Industries Ltd.).

## Author Contributions

Y.K. and M.K.K. performed the research and wrote the paper.
